# Current Progress in the Science of Novel Adjuvant Nano-Vaccine-Induced Protective Immune Responses

**DOI:** 10.3390/pathogens13060441

**Published:** 2024-05-23

**Authors:** Mansab Ali Saleemi, Yan Zhang, Guoquan Zhang

**Affiliations:** Department of Molecular Microbiology and Immunology, College of Sciences, University of Texas at San Antonio, San Antonio, TX 78249, USA; mansab.saleemi@utsa.edu (M.A.S.); yan.zhang@utsa.edu (Y.Z.)

**Keywords:** vaccination, adjuvants, delivery systems, immune response, future perspectives

## Abstract

Vaccinations are vital as they protect us from various illness-causing agents. Despite all the advancements in vaccine-related research, developing improved and safer vaccines against devastating infectious diseases including Ebola, tuberculosis and acquired immune deficiency syndrome (AIDS) remains a significant challenge. In addition, some of the current human vaccines can cause adverse reactions in some individuals, which limits their use for massive vaccination program. Therefore, it is necessary to design optimal vaccine candidates that can elicit appropriate immune responses but do not induce side effects. Subunit vaccines are relatively safe for the vaccination of humans, but they are unable to trigger an optimal protective immune response without an adjuvant. Although different types of adjuvants have been used for the formulation of vaccines to fight pathogens that have high antigenic diversity, due to the toxicity and safety issues associated with human-specific adjuvants, there are only a few adjuvants that have been approved for the formulation of human vaccines. Recently, nanoparticles (NPs) have gain specific attention and are commonly used as adjuvants for vaccine development as well as for drug delivery due to their excellent immune modulation properties. This review will focus on the current state of adjuvants in vaccine development, the mechanisms of human-compatible adjuvants and future research directions. We hope this review will provide valuable information to discovery novel adjuvants and drug delivery systems for developing novel vaccines and treatments.

## 1. Introduction

Vaccination has been one of the most significant achievements in the history of human medicine. Particularly, through vaccination, we were able to eradicate smallpox and rinderpest as well as significantly reduced the morbidity and mortality of many devastating infectious diseases in the past two centuries. However, in the last two decades, we have also experienced various infectious disease outbreaks, such as the severe acute respiratory syndrome (SARS) pandemic in 2003, the Ebola outbreak in 2014, the Nipah epidemic in India in 2018 and the most recent COVID-19 pandemic starting in late 2019 in China [1,2,3]. The Ebola outbreak in 2014 caused a serious crisis among developed nations; in particular, there was a very high mortality rate for the Ebola virus as it spread in West African countries [2]. The emergence of new variant strains of the Ebola virus and SARS virus has alerted the scientific community to explore new technologies to develop more effective vaccines against the potential re-emerging infectious diseases [4]. Additionally, there is an urgent need to develop safe and effective vaccines against tuberculosis and AIDS [5].

Adjuvants are substances that can be formulate with vaccine antigens to enhance and modulate the immunogenicity of the antigen through various mechanisms [6]. Their usefulness has been demonstrated for inactivated, subunit and recombinant protein antigens via different actions; the most notable functions are their abilities to serve as carriers, depots and stimulators of targeted immune responses. Aluminum was the first adjuvant that was used in the formulation of human vaccines based on its ability to stimulate an enhanced level of antibody production [7]. However, aluminum not only fails to elicit robust cellular immune responses but also possesses the potential risk of developing long-term inflammation in the brain [8]. In addition, some adjuvants can cause local and systemic toxicity in immunized hosts [8]. It has been reported that Quil A in Freund’s adjuvant can induce local toxicity [9]. On the other hand, adjuvants based on the pathogen associated molecular patterns (PAMPs) contain microbial molecules that trigger inflammation and dendritic cell maturation through pattern recognition receptors [9]. Another adjuvant was completely formulated by Freund’s using mineral-oil-based emulsions containing heat-killed mycobacteria [10]. Despite the potential of adjuvants to stimulate local immune reactions, they failed to generate a strong cell-mediated immune response, which demanded the development of new adjuvants for successful vaccine delivery [9]. According to a web-based central database for vaccine adjuvants, over a hundred vaccine adjuvants have been utilized against various pathogens, though only a few have been licensed for human use [11].

Nanoparticles (NPs) have gained widespread attention as delivery vehicles for human vaccines [12]. Nano-vaccine formulations can facilitate prolonged drug release and targeted delivery in addition to providing enhanced immunogenicity and stability [12]. NP-based vaccines with different properties have been approved for human use [12,13]. The utilization of nanoparticle-based delivery methods aims to enhance the duration of the antigen presentation cell (APC)- and dendritic cell (DC)-mediated uptake process, which results in the stimulation of DCs and facilitates the cross-presentation of the antigens [12,13]. NPs help in preventing the degradation of adjuvants and antigens by proteolytic and enzymatic processes [14]. A vaccine can be delivered to a target site by either enclosing it inside the NPs or coating it on their surface [15]. The ability of an NP-based delivery system to load multiple components in a single carrier enables a prolonged, targeted and simultaneous delivery of various antigens, DNA plasmids, toxins and adjuvants [15,16]. Various factors are taken into consideration when developing vaccines, such as the composition of the vaccine, its effectiveness and low immunogenicity [17,18,19]. The unique physical attributes of NPs, such as their shape, size and surface charge, make them ideal delivery vehicles for vaccines [20]. NPs can be surface engineered with different proteins, peptides, polymers and other targeting agents, which makes them a versatile delivery vehicle for nano-vaccine formulations [20]. The design of NP-based vaccines may be utilized to develop multimodal imaging that enables the visualization of a vaccine in the host cells, which can improve therapeutic efficacy [20,21]. Although NPs have benefits, they also have some limitations, such as an undesirable interaction with the reticuloendothelial system (RES) and a lack of colloidal stability in physiological conditions because of protein corona formations [22,23].

However, a biomimetic NP exhibits enhanced stability and is designed to avoid interactions with immune cells [24,25]. In general, nano-vaccines are developed by utilizing carrier NPs that mimic biological membranes [26]. Nano-vaccines are then administered into the host cells to promote prolonged immune evasion and circulation [26]. For instance, biomimetic liposomes are created by the dispersion of lipids in water [27]. Biomimetic liposomes have a high loading capacity and are capable of delivering either hydrophilic or hydrophobic drugs [28]. A cell-membrane-coated NP is a biomimetic nanocarrier that has a core–shell structure [28]. This core–shell structure is formed by the NP’s hydrophobic core and a layer of membrane coating [28]. Various cell membranes are utilized to cloak synthetic NPs, which are then subjected to a top-down fabrication process [29]. This method ensures that the core NPs retain their physicochemical properties while maintaining their cellular composition [29]. In one study, membrane-coated NPs were studied where red blood cell (RBC) membranes were extruded onto a polymeric NP [28]. Self-assembling proteins are known to have high stability and symmetry, which allows them to be arranged into smaller particles that are about 10–150 nm in size [30,31]. These polymeric NPs play different roles in the body, and are mainly utilized as carriers of vaccines due to their ability to assemble into structures that look similar to a microbe’s natural architecture [32]. In this review, we discuss the current state of adjuvants in vaccine development, mechanisms of human-compatible adjuvants, and recently Food and Drug Administration (FDA) approved nano-vaccine adjuvants as well as current challenges and future developments.

## 2. Adjuvanticity of Nanoparticles

Adjuvants are substances that stimulate the immune system in a non-specific manner against the antigens. Aluminum salts, oil emulsions, non-ionic block copolymers and various other types of adjuvants are commonly used as immune-stimulating agents, derivatized versions of polysaccharides, liposomes and bacterial derivatives [6,33]. Aluminum-based compounds are widely used in vaccines as adjuvants, which are approved for use in humans [34]. However, these adjuvants have various limitations, including their low effectiveness in producing peptide vaccines and allergic reactions in the injection site [35,36]. Due to these limitations, the production of versatile and effective adjuvants is essential. Studies have shown that the effectiveness of NPs for stimulating the immune system is comparable to that of aluminum-based products [37]. Although the exact mechanisms of the adjuvanticity of NPs are not known, various explanations have been presented for this phenomenon [38]. Due to their size and structural features, NPs can easily replicate the effects of a bacterial or viral infection [16]. These nanoparticles can be ingested by APCs, which can stimulate the immunological responses [16]. According to one theory, NPs can stimulate the development of a cellular immune response by interacting with CD8^+^ dendritic cells [39]. A characteristic of NPs is that they can transfer from the subcutaneous tissues to the lymph nodes when they are less than 100 nm in size [39]. A stimulated innate immune response will result from the antigen being delivered to mature immune cells in these nodes [39]. According to the study, the use of NPs in a vaccine can increase the antibody production [40]. The finding supports the idea that the use of these substances can improve the effectiveness of vaccines [38,40]. Lately, nanomedicine and materials science communities have developed lipid nanoparticles (LNPs) as carriers for small molecule delivery, and these particles are now a vital component of COVID-19 mRNA vaccines [41]. LNPs are well positioned to play a significant role in the developing field of genomic therapies, where safe and effective in vivo delivery has been a key challenge. In most circumstances, LNPs could address the limitations of other methods of delivery system to the host cells. For instance, viral vectors are often very effective in delivering genes to the nucleus, but their payload size limitations and a restricted redosing potential could limit their use [41]. Table 1 presents an overview of the advantages and disadvantages of using different nanoparticles in the development of vaccines.

The development of antibodies that target either nanoparticles or antigens is involved in the response of the immune system to nano-vaccines. No specific immune response was reported in the immunization of either cationic dendrimers or nanoparticles made up of fullerene [55]. According to reports, the NPs serve as a delivery method for a bystander vaccination but are not themselves antigenic [55,56]. This is particularly important since the efficacy of particle-based vaccines can be affected by the development of antibodies that respond to the NPs. There are only a few studies suggesting that certain NPs may become antigenic after being linked to carrier proteins [55,56]. It is important to note that nanosystems can enhance the capacity of immunostimulatory molecules by allowing them to be co-encapsulated. Various compounds, including dextran NPs and chitosan nanocapsules, are known to interact with receptor agonists such as CpG or imiquimod [57]. In one study, researchers modified CpG with a reactive thiophosphate group which allows coupling to the cysteamine functionalized NPs [57]. They reported that the thiophosphate terminal group of CpG and the functionalized NP exchange disulfides to cause this reaction to happen. The covalent bond can be removed by using 2-mercaptoethanol to simulate the reducing environment of the cell’s endosomal compartment. Another study designs a prolong release nanosystem with imiquimod-loaded chitosan-coated polymeric nanoparticles (CS-IMQ NPs) for an effective drug delivery application [58]. The results show that the zeta potentials of the CS-IMQ NPs have shifted to positive values, indicating that the amino groups on the CS molecule are located on the surface of NPs. Moreover, the ionic interactions between the positively charged CS-coated IMQ NPs and the negatively charged cell membrane could prolong the residence time [58]. However, electrostatic and hydrogen bonding interactions are involved in the adsorption of CS onto the surface of polymeric NPs. The recognition of nano-vaccines by specific receptor molecules can improve their activation and also strengthen the response of the immune system [59,60].

**Figure 1:**
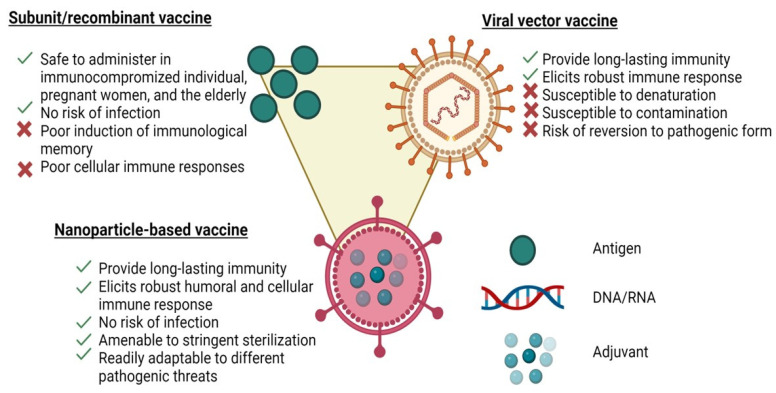
Schematics of a representative nanoparticle vaccine, a subunit vaccine and a viral vaccine illustrating the advantages and disadvantages of each platform.

The advancements in materials engineering over the past decade have led to new directions in developing vaccines. In particular, vaccines have been extensively developed using synthetic nanoparticles [61]. In vitro studies have shown that synthetic nanoparticles of 25 to 200 nanometres in diameter could stimulate the development of a strong immune response against antigen targets [61]. The safety profile of synthetic nanoparticle vaccine candidates is better than that of attenuated ones due to their non-replication properties [61]. Many of the advantages of nanoparticle vaccines can be attributed to their resemblance to natural viruses, as shown in Figure 1. However, the use of nanoparticles such as synthetic polymer-based NPs, liposomes or metal-based NPs is limited due to their low stability, structural heterogeneity, potential immunogenicity, high toxicity and off-target activity [62]. Therefore, alternative protein-based NPs such as virus-like particles (VLPs) are required to overcome these constraints. Protein-based NPs have several benefits including their ease of functionalization, controlled assembly, biocompatibility and biodegradability [62,63]. Various viral features, such as the nanoscale morphology, multivalent antigen presentation and controlled antigen/adjuvant delivery, can stimulate the immune responses at cellular and physiological levels. It should not come as a surprise that nanoformulations adopting virus-like properties can be more potent than traditional subunit formulations because the human immune system has not developed to respond effectively to infectious viral nanoparticles (Figure 1) [64]. In addition, the human immune system has been geared to pathogens including viruses and it may also respond to nanoparticles.

## 3. Advancement in Novel Adjuvant Development

The development of new adjuvants will require the establishment of a new discovery pipeline focused on the identification and evaluation of end-stage immunological activities. With the identification of various immune pathways and agonists, the development of new adjuvants can now focus on determining their phenotypic responses. We will discuss the different aspects of adjuvanticity that are most important to engineer.

### 3.1. Formulation of Next-Generation Adjuvant for Specific Targeted Delivery

A next-generation adjuvant is often developed to improve the efficacy and delivery of immunomodulators. The early approved adjuvants mainly targeted non-interest cells using heterogenous and suspension-based strategies [65]. These strategies are typically not applicable to recently approved adjuvants. However, the development of novel and improved vaccines against re-emerging infections has been made possible by the discovery of new combinations of adjuvants such as adjuvant systems (ASs) [66]. The idea for adjuvant systems originated by serendipity [66]. ASs consist of combinations of immunostimulatory molecules designed to improve and increase protection through vaccinations over traditional formulations that incorporate aluminum salts. The AS series of adjuvants from GlaxoSmithKline (GSK) contains various components that are designed to form nanostructures [66]. Another example is the Matrix-M adjuvant from NovaVax, which combines cholesterol and phospholipids with immunostimulatory saponins [67,68]. As the complexity of adjuvants continues to increase, it is important that the formulation be considered early in the discovery and development phase. Adjuvants must be transported to various subcellular sites to facilitate receptor binding, as the diversity of these substances continues to expand. Endosomes, cytosol and outer membranes are potential target receptors [68]. Formulations can utilize these locations to distribute adjuvants. For instance, the STING adjuvant is an innate receptor that is found in the cytoplasm, while cationic lipid nanoparticles dramatically enhance endosomal escape [69]. The size of nanoparticles is a crucial factor to consider when it comes to controlling their efficiency in lymphatic drainage [70]. For example, the nanoparticle size of 10 to 100 nm is ideal for facilitating the easy diffusion of key secondary lymph organs [70,71]. The nanoparticle size of 50 nm plays a vital role in the activation of inflammasome reactions as well as endosomal escape [72]. The reduction in cellular uptake caused by larger nanoparticles can limit their effectiveness [73].

The use of external targeting ligands can help facilitate the delivery of desired cellular types. Although most of the adjuvant receptor’s activity is concentrated on immune cells, they can also be affected by the bystander tissues [74]. The activation of these cells can promote the development of proinflammatory conditions linked to atherosclerosis due to the expression of TLR4 on arterial smooth muscle [74]. Therefore, off-target effects on bystander cells should be minimized. Targeting ligands not only lessens side effects but also boosts effectiveness. This strategy is mainly utilized to target certain cell types, such as DC-SIGN and dendritic cells [75]. However, it is believed that targeted delivery could benefit other niche cell types [75,76]. For instance, stromal and lymphatic endothelial cells are known to be involved in long-term antigen storage [77,78]. The PRG2 and DAP12 receptors can be utilized to target first responders’ cells, which are known to boost the phagocytosis of microstructures [79,80]. Due to the increasing understanding of innate immunity, the formulation of adjuvants is expected to play a more prominent role in directing therapeutic responses.

### 3.2. Relatedness of Signal Engineering and Target Identification

Selectively altering the signaling pathways in order to elicit desired immune reactions can be beneficial for adjuvants. Instead of developing more potent receptor agonists, it is believed that drugging other parts of the innate immune system could yield more diverse outcomes [81]. Besides pathogen recognition receptors (PRRs), various cellular signals, such as proliferation and metabolism, can also be associated with immune responses [82]. The activation of innate pathway leads to the formation of fate-related cytokines that control the polarization of T-cells [82]. The effects of adjuvants on the elicited cytokines can have a significant impact on the adaptive responses of the human body. Vaccine reactions can be triggered by the actions of certain cytokines. Reducing the cytokines that are linked to inflammation could help minimize the adverse effects of this condition and improve the likelihood of successful clinical translation [82]. Therefore, it is believed that exploring new routes and modifying targeted pathways can help address the various signaling problems that can arise due to innate factors.

#### 3.2.1. Possible Strategies for Adjuvanticity

**Figure 2:**
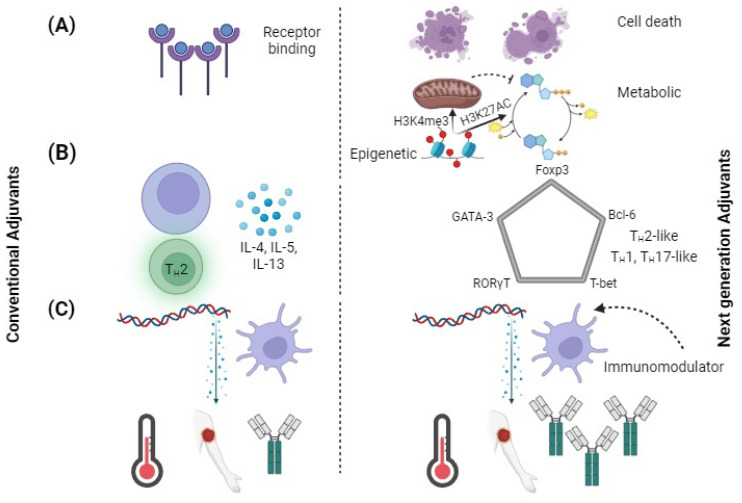
Using adjuvants to engineer new immunological effects. Comparison of conventional adjuvants to next-generation adjuvants. (**A**) Targeting pathways of next-generation pathway other than just PRRs. DAMPs are released by cell death, metabolic changes increase cell sensitivity, and epigenetic changes reprogram cells to adopt a trained immune phenotype. (**B**) T-cell polarization shifted from classifying cytokines to identifying transcription factors. Next-generation adjuvants could generate immunophenotypes. (**C**) Conventional adjuvants elicit large amounts of inflammatory cytokines, whereas next-generation adjuvants decrease reactogenicity and increase adaptive responses.

Adjuvant targets should expand beyond the ligands that are commonly used for targeting toll-like receptors (TLRs) [83]. Over the past decade, various research studies have discovered new receptors for pattern recognition such as cytosolic sensor STING [83,84]. Various cyclic dinucleotides, such as cyclic guanosine adenosine monophosphate (cGAMP), are known to be STING ligands and could be used as adjuvants [83,84]. In addition, the NLRP3 innate defense mechanism can address broad changes in the homeostasis by recognizing various damage-associated molecular patterns (DAMPs) and pathogen-associated molecular patterns (PAMPs) [84]. Besides PRRs, other signaling pathways have an impact on innate immune activation and could be used for developing future adjuvants (Figure 2A). The development of cell death pathways through the combination of pyroptosis, apoptosis and necroptosis can result in enhanced cross-presentation by adjacent cells [85,86]. The activation of metabolic and epigenetic pathways by promoting gene activation through H3K4me3 and H3K27AC are altered in trained immunity [87]. Trained immunity is a recently developed concept in which innate immune cells show “memory” by increased activation with re-exposure to even heterologous pathogens [87]. The pathways are a vast resource of new adjuvants, as numerous activators of these routes are being discovered.

#### 3.2.2. Adjuvant Reactogenicity Reduction

The approval of new adjuvants can be challenging due to reactogenicity [88]. While providing enough stimulation, adjuvants must also avoid excessive activation, resulting in various side effects. The inability to balance the number of adjuvants in vaccines has a negative effect on the clinical approval of these substances. The effects of adjuvant-associated systemic inflammation could include fever, headache and injection site pain [9]. After administration, inflammation can be monitored by measuring the levels of pro-inflammatory cytokines, such as interleukin-6 (IL-6) and tumour necrosis factor-alpha (TNF-α) [89]. Besides individual patients, severe reactogenicity can also affect the perception of vaccinations among population [89]. Next-generation adjuvants should focus on minimizing the reactogenicity of vaccines while maintaining their efficacy.

Early in the discovery phase, it is important to determine the reactogenicity of lead compounds to ensure that they are identified and tested for their potential to cause inflammation or toxicity [90]. Some messenger ribonucleic acid (mRNA) and lipid nanoparticles cause excessive inflammation; however, reactogenicity may be able to control the doses of mRNA vaccines [90]. The reactogenic properties of mRNA are currently addressed through a nucleoside-modified form of mRNA [91]. It is not clear how lipid nanoparticle therapy can activate innate immunity. Various theories have been presented regarding the possibility of detecting particle-caused cellular damage [91]. Identifying the innate immune system’s mechanisms is very important in developing effective adjuvants. A combination of appropriate adjuvants and lipid nanoparticle vaccine systems could be used to treat various diseases, such as Lyme disease and COVID-19 [92,93]. For instance, saponins as adjuvants are used in several recently authorized vaccinations [94,95]. These plant-derived small compounds have powerful immunostimulatory properties, but also cause a cytotoxic effect which limits the effective dose of these adjuvants [96].

However, researchers have been utilizing immunomodulators to treat inflammation associated with adjuvanticity [97,98]. These immunomodulators could enhance the level of antigen-specific antibodies in a vaccine. They are also combined with PRR agonists to decrease inflammation (Figure 2C). The effect is applicable to different types of PRR antigens and PRR agonists. The immunomodulating agents are responsible for targeting intermediate signals that are downstream of the receptor [98]. Various molecules that can modify the receptor pattern recognition pathway in response to antigens could be used to reduce the reactogenic effect of vaccines in the future.

#### 3.2.3. Polarization of T_H_ Cells

The action of an adjuvant could influence the development of adaptive immune responses. The innate immune system secretes a variety of fate-related cytokines [99]. These are known to drive the polarizing effector state of CD4^+^ T cells [99]. Although adjuvants can stimulate the polarization of T-cells by delivering specific cytokines, their use is restricted. A number of adjuvants, such as CpG 1018, are known to induce T_H_1 in patients; however, alum is a powerful T_H_2 inducer [100,101]. Interferon gamma (IFN-γ), tumour necrosis factor-alpha (TNF-α) and interleukin-2 (IL-2) are secreted by Type 1 T helper (T_H_1) CD4^+^ cells to support cell-mediated immune responses [102]. IL-4, IL-5 and IL-13 are secreted by T_H_2 CD4^+^ cells to stimulate antibody responses. Prophylactic vaccines are typically designed to trigger T_H_1 responses but, ideally, vaccines and immunotherapies might be tailored for different applications. Alternative T_H_ polarization can be beneficial for certain applications that are not related to the spread of infectious diseases. The production of IL-17 by T_H_17 cells is known to protect against various types of bacteria [103]. They also play a role in the response of a host cell to the infections caused by *Salmonella*, *Klebsiella* and *Pseudomonas* [103]. The presence of T_H_17 cells could help protect against tuberculosis (TB) by inducing inflammation and neutrophil recruitment [104]. The extent to which the polarization of T_H_17 is maintained is also important, as overproduction of this response may lead to more severe tuberculosis pathologies [105]. Therefore, specific control of T_H_ polarization is required rather than just general shifts from one to another bias. In the current state of adjuvant development, downstream effects such as T_H_ polarization are considered only after signaling processes have been initiated by receptor–ligand interactions [105]. The paradigm for developing new adjuvants may be changed by the discovery of next-generation agents, specifically those that target end-stage immunophenotypes, such as T_H_ polarization.

The conventional nomenclature for T_H_ polarization may be restricted with better understanding of the helper T-cell compartment [106]. Traditionally, helper T cell secreted cytokine profiles have been used to determine T_H_ polarization. CD4^+^ T cells that play distinct roles, such as the T-cells (Tregs) and follicular helper T-cells (T_FH_), can complicate the classification of T_H_ [107]. In the past few years, various T_H_ subtypes, such as T_H_9, T_H_22 and T_H_GM, have been proposed [107,108]. The current classification system is imprecise due to the level of cytokines that may be expressed. However, the proposed adjuvant classification of T_H_ polarization based on the five master transcription factors T-bet, GATA-3, RORT, Bcl-6 and FoxP3 is shown in Figure 2B. Earlier studies suggested that these transcription factors’ expression was mutually exclusive; however, more recent research indicates that co-expression of transcription factors is conceivable [109]. T_H_ polarization can be considered as a component of a multidimensional space and transcription factor levels due to its axis variables. New vaccine development could be achieved by utilizing adjuvants that can polarize the responses of T_H_ to different environments.

## 4. Molecular Target of Adjuvant Nano-Vaccine

An adjuvant can have numerous advantages, such as diminishing the antigen quantity in each vaccine dose, increasing the stability of the component and reducing the number of vaccination attempts. These factors can help improve the immunogenic power of the vaccines [6]. A wide variety of adjuvants has been developed for use in the production of vaccines (Table 2) [110].

**Figure 3:**
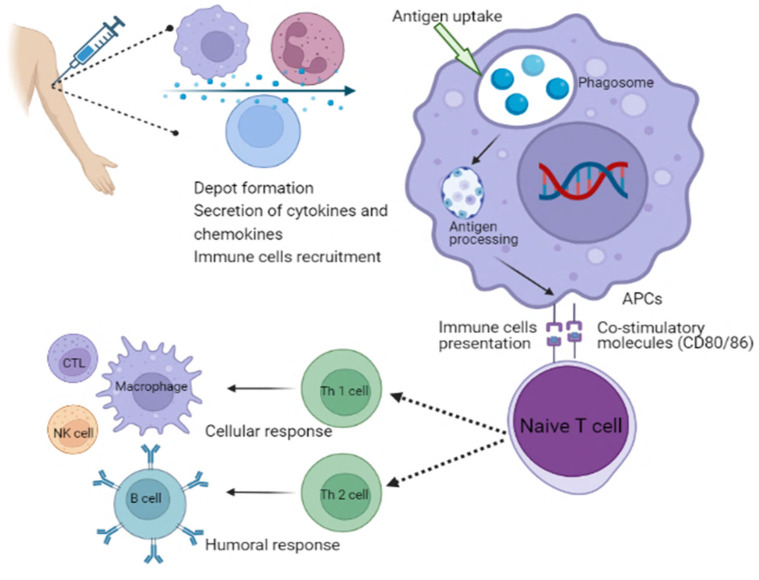
Schematic illustration of the mechanism of action of adjuvants involves in the activation of immune system. APCs, antigen-presenting cells; NK cell, natural killer cell; CTL, cytotoxic T lymphocytes; Th, T helper cells.

Different types of adjuvants can be grouped depending on their attributes, such as origin, mechanisms of action or physicochemical properties [127]. Adjuvants are classified based on their mechanisms of action. Adjuvants are typically divided into two main groups: delivery systems and immune potentiators [128]. A delivery system’s adjuvant can be associated with the utilization of antigens, which makes it an effective antigen carrier. For instance, the translocated antigen is inherently subject to conformational constraints due to the presence of a transporter that facilitates antigen export to the cytosol [129]. Antigens are anticipated to go through an unfolding step prior to translocation because it is unlikely to be transferred in their native structure considering the narrow diameter of known transporter pores [129]. This theory is supported by the fact that structurally flexible protein translocates into the cytosol more efficiently than rigid protein. Furthermore, GILT, a gamma interferon-inducible lysosomal thiol reductase constitutively expressed in APCs, reduces disulfide bonds during unfolding, which is necessary for the cytosolic export of viral disulfide-rich antigens and subsequent cross-presentation [129]. These therapies can stimulate the development of a local inflammatory response by priming the body’s innate immune system. This process results in the activation of immune cells that can be utilized at the injection site [130]. Th polarization is the process by which microbial ligands engage different PRRs, such as toll-like receptors, to activate DCs [131]. This results in the release of cytokines that regulate the development of naive effector CD4^+^ Th cells into distinct functional subsets, while DCs that produce IL-1b, IL-6, IL-23 and TGF-b stimulate Th17 responses which are crucial for immunity against extracellular protein. However, those that produce IL-12p70 and express the costimulatory molecules CD40, CD80 and CD86 stimulate Th1 responses for combating intracellular protein in the cytosol [131]. Moreover, DCs that produce TGF-b and IL-10 stimulate Treg responses in the absence of appropriate costimulation, whereas DCs that produce IL-10 in addition to different costimulatory molecules (CD80, CD86, CD40 and OX40L) stimulate Th2 cells for the control of extracellular protein. Tregs play an important role in the regulation of Th1, Th2 and Th17 responses, whereas T cells themselves express specific master lineage regulators that are responsible for the relevant Th responses (Figure 3) [131]. Adjuvants are useful in helping to enhance the effectiveness of vaccines for the elderly individuals due to the immunosenescence phenomenon, which is a reduction in the response of immune system to natural or artificial stimuli [131]. In this case, the use of adjuvants may be considered as a tool to overcome the limitations of vaccines. For example, adjuvants can be helpful in boosting the response of certain subunit vaccines, that are typically too weak to stimulate an active immune response on their own [132]. Vaccines that are licensed for use against meningococcal disease do not contain adjuvants [133]. This is because the protein carrier in the vaccine’s conjugation helps in the stimulation of immune response [133]. New molecules and factors that have enhanced adjuvant capabilities should be strengthened through the expansion of studies in in vitro and in vivo platforms. The approval of new drugs may be delayed or cancelled due to various factors, such as the use of new cellular technologies, the use of adjuvants, or the application for transfers or process modifications. This aspect can hinder innovations, increase costs and delay the availability of vaccines in countries with limited resources [133].

The COVID-19 pandemic emphasized the importance of having the proper vaccines in order to prevent a new outbreak. Vaccines for COVID-19 are known to have intrinsic properties that are linked to the liposomes and serve as RNA carriers [110]. Although the newest COVID-19 vaccine uses a standard platform, it is also equipped with an adjuvant called Matrix-M, which is made up of *Quillaja saponaria* extracts [110]. Safety is one of several factors to be taken into account when selecting an adjuvant. An ideal adjuvant is needed that is easy to produce and safe to handle. It must also have good pharmaceutical characteristics, such as pH, endotoxin levels and osmolality as well as lasting a long time [134]. It could be challenging to respect the various properties of the vaccines without affecting its safety. Therefore, it is very rare to add vaccine adjuvants to the current vaccines. Despite the advancements that have been made with vaccines, there are still concerns about their safety. Compared to other substances, adjuvants have always been associated with increasing concerns about safety among members of the scientific community [134]. At present, vaccines do not typically cause permanent adverse effects, such as local erythema and injection site reactions, flu-like sensations and mild general malaise. These adverse effects of vaccines subside within a few days or hours following vaccination, although anaphylaxis or other severe reactions have been occasionally reported after vaccinations [135].

## 5. Delivery of Nanoparticle-Based Adjuvants through Microneedles

In recent years, there has been a growing interest in microneedle-based techniques among the many strategies developed for antigen delivery through the skin [136]. Because of their minimal penetration depth, microneedles have the primary benefit of being able to pierce the skin in a minimally invasive manner and deliver their payload in the superficial skin layers potentially without causing pain [137]. Various types of microneedles have been developed for the delivery of vaccines [136]. These include coated and dissolving microneedles, which may penetrate the skin and release a dry antigen into the dermis and epidermis. On the other hand, antigens or particulate formulations can be applied topically as suspensions or solutions using hollow microneedles. For this purpose, researchers developed a hollow microneedle device using etched fused-silica capillary-based microneedles, enabling accurate and controlled injections into the dermis and epidermis [138,139]. Comparing hollow microneedles to coated or dissolving microneedles, the advantage is that changing the dosage, formulation or administration depth takes less time. Studying the optimization of vaccination formulations or parameters—such as vaccine dose or penetration depth—is especially beneficial in this context.

Most studies on vaccination have used intramuscular or subcutaneous injections; however, no studies have directly examined the effectiveness of various nanoparticles for the topical administration of vaccines [138,139,140]. In one study, researchers evaluated the ability of co-encapsulated adjuvant or antigen-loaded nanoparticles to stimulate humoral and cellular immune responses following intradermal immunization using hollow microneedles [140]. They designed four distinct nanoparticulate delivery systems, namely liposomes, gelatin nanoparticles (GNPs), mesoporous silica nanoparticles (MSNs) and poly (lactic-co-glycolic) acid (PLGA) nanoparticles [140]. Each of these systems has different physicochemical characteristics. Numerous studies have been conducted on PLGA nanoparticles and liposomes as biocompatible and biodegradable nanoparticle vaccine delivery systems [141,142,143]. MSNs gain increasing attention for use in vaccine delivery due to their high loading capacity, excellent in vivo biocompatibility, and controlled size and mesostructure [144]. However, gelatin-based nanoparticles have been investigated as potential vaccine carriers because of their excellent biocompatibility, durability and suitability for surface modification [145].

Previously, inactivated viral vaccinations were administered using an internal hollow microneedle/applicator device [136,138,139]. Most of the studies used hollow microneedles with a 20 μm bore diameter [140], although larger-sized nanoparticles (> 100 nm) have not been employed with this technology. These pilot investigations demonstrated that, if the bore diameter was 20 μm, the hollow microneedles could be blocked as a result of occasional nanoparticle aggregation [140]. This problem could be resolved by increasing the bore diameter to 50 μm since a larger bore diameter reduces particle obstruction in the system. Consequently, there was neither a blockage nor a leakage of formulation during the vaccination studies. After every injection, a bleb formed at the injection site, indicating that the intradermal injection was successful. Moreover, no adverse side effects, including erythema or skin induration, were noticed at the injection site during experimental study [140]. The results also show that the type of nanoparticle greatly influences the responses, for instance, OVA encapsulation and co-encapsulation with poly(I:C) produced a notably greater IgG2a antibody response than the OVA/poly(I:C) solution [140]. PLGA nanoparticles, and specifically cationic liposomes, were shown to elicit the highest IgG2a, CD8^+^ T cells and CD4^+^ T cells responses, indicating their superiority when it comes to intradermal vaccination [140]. The in-house-designed hollow microneedle/applicator device is a great tool for screening various intradermal vaccine formulations and administering intradermal vaccinations based on nanoparticles.

## 6. Promising Strategy for Antigen Self-Presentation and Immunosuppression Reversal by Nano-Vaccine

### 6.1. T-Cell Immunotherapy

T-cells that are antigen-specific are required for the successful development of immunity in the body [146]. The process involves the interaction between antigen-presenting cells and T cells. The ability to control the APC function is a crucial step in developing therapeutic strategies that involve the use of T-cell therapy. It has been proven that the activation of CD8^+^ cytotoxic T lymphocytes (CTLs) is a crucial factor in immunotherapy. The composition of tumour-derived peptide and the major MHC-I molecules used by APCs in the process determine the development of T cells [147,148]. Vaccines that contain protein and peptide components rely on random interactions with APCs. Inappropriate encounters could suppress an immunological response [149]. Efficient antigen presentation is a critical factor that contributes to the activation of CD8^+^ T cells. For instance, the shortcomings of cancer vaccines can potentially be explained by the two circumstances. The ability to customize the activation program and cell-based therapeutic agents is the most advantageous feature of adoptive cell therapies. For dendritic cell (DC)-based cancer vaccine therapy, it is possible to generate an endogenous CTL response that is modulated by a standard MHC-I restriction [150,151]. It is also inevitable for the activated DCs to remain in the body for a brief period after injection. This will eventually lead to a small number of them moving to the lymph nodes’ draining areas [152]. In spite of the progress that haematological malignancies have made, the use of T-cell therapy has not been successful in treating solid tumours [153].

In addition, immune checkpoints can hinder the development of CTL responses in the tumour’s immune microenvironment [154]. Despite the immense potential of immune checkpoint therapy, only a small number of patients are able to achieve complete responses. This is because they lack a sufficient pre-existing cytotoxic T-cell response [155,156]. Combination therapy involving the use of immune checkpoint inhibitors and a robust CD8^+^ cell response is ideal. The exact mechanisms by which checkpoint blockades work are still not known. Studies show that the CD28 pathway is necessary for the rescue of CD8^+^ cells. It also suggested that the CD28/B7 pathway is a crucial factor in the treatment of cancer patients using programmed cell death protein 1 (PD-1) therapy [157]. It has been hypothesized that a combination of B7-1 and B7-2 costimulatory molecules, as well as a PD-1 antibody, could be used to overcome the challenges of immunotherapy [158]. However, the biomimetic process involves the synthesis and presentation of protein cargos onto a cell’s surface, which represents a promising strategy for producing nanocarriers with ligands [159,160]. The process of biosynthesis can maintain the structural and functional properties of a protein [161].

### 6.2. Immunotherapy Using PD-L1-Targeting Nanocarriers

Immune cells normally identify and eradicate cancer cells as foreign pathogens. Unfortunately, tumours have the ability to impede cellular signaling and metabolism through either increased immune cell signaling inhibition or enhanced immune receptor inhibition on the tumour surface [162]. Immune checkpoint blockade therapies have demonstrated notable clinical success, particularly when it comes to targeting the PD-ligand-1 (PD-L1) and programmed cell death protein-1 receptor (PD-1) axis [163]. Scientists are currently employing medical treatments, instruments and devices based on nanotechnology to enhance this therapy’s efficacy, safety, sensitivity and customization [164].

In addition to peptides that target PD-1, PD-L1 is a possible target. Researchers coupled a peptide that recognizes PD-L1 to an affinity peptide for tumour vasculature, which was subsequently added to methoxy poly(-ethylene glycol)_3000_-poly(ε-caprolactone)_20000_(MPEG-PCL) nanoparticles. The nanosystem treatment group survival time was increased by 47.5 days and 19.5 days, respectively, for tumour-bearing mice when treated with the paclitaxel-loaded NP [165]. In another study, IR780 was coupled with an anti-PD-L1 peptide to produce NPs [166]. When the tumour was exposed to laser radiation, the nanosystem was able to efficiently accumulate at the site of the tumour and eliminate it. Peptide concentrations may also affect CD8^+^ T cell, CD4^+^ T cell and Treg populations. This could result in the restoration of tumour microenvironment (TME) homeostasis, which strengthens the immune system’s ability to inhibit the growth of tumours [166]. This animal model showed decreased tumour growth in conjunction with increased effector T cells and immune-activated cytokines such IL-6, IL-2, TNF-α and IFN-γ [166]. In one study, researchers screened for PD-L1-binding peptides before fabricating NP [167]. Analysis of homology and structure showed that PD-L1 interacted with either PD-L1 Pep-1 or PD-L1 Pep-2. Mice that received injections of PD-L1 Pep-1 and PD-L1 Pep-2 showed decreased tumour growth and an elevated CD8^+^/FoxP3^+^ ratio; however, when peptides and doxorubicin were delivered together via liposomes, the tumour was totally eradicated and the percentage of CD8^+^ T cells increased dramatically [167]. On the other hand, human serum albumin (HAS)-curcumin NPs were coated by one group of researchers using a PD-L1 binding peptide (TYLCGAISLAPKAQIKASL) [168]. Peptide-HAS-curcumin NPs were efficiently internalized into PD-L1-expressing breast cancer cells, with a size of 246 nm. A controlled in vivo investigation should be carried out to validate the outcomes of this strategy, even though the nanosystem increased the death of cancer cells [168]. Another group of researchers conjugated a PD-L1-binding peptide with two hydrophobic stearyl chains using a pH-sensitive linker to show possible chemo-immunotherapy efficacy [169]. This newly discovered compound combines with doxorubicin to generate a complex. The NPs disintegrate in the acidic tumour microenvironment to release doxorubicin, which triggers both immunotherapy and chemotherapy at the same time [169]. This combined strategy both in vitro and in vivo strongly suppressed CT26 tumours and induced an immune response.

## 7. FDA Approved Nano-Vaccine Adjuvant for Clinical Trial

Due to the safety concerns associated with the development of new vaccine adjuvants, many of them have been thoroughly evaluated in both clinical and preclinical studies. An adjuvant commonly used in the United States, namely alum, has been approved. Over 70 years ago, the first vaccine that used alum adjuvant was approved. In 1997, the European Commission permitted the use of a modified influenza vaccine formulation that contained the adjuvant MF59. In 2009, a vaccine adjuvant known as AS03 was licensed for use in the influenza vaccine. The use of liposomal adjuvants, such as virosomes, was allowed by the FDA in the vaccines for influenza and hepatitis A in 2000. In addition, a combination of the alum-based adjuvant and monophosphoryl lipid A (MPLA) adjuvant, namely AS04, has been granted approval in the US and Europe. Table 3 shows the various adjuvants that are licensed to be used in humans [170]. In spite of having various advantages, such as lower cost, better safety and high yield, many vaccine antigens are not very effective at stimulating the body’s immune response due to the lack of certain intrinsic factors, such as PAMPs. Understanding the role of PAMPs in stimulating the immune response and how this process is regulated and coordinated is very important [171]. It is stated that particulate materials can stimulate the innate immune system’s signals [172]. Adjuvants were developed to stimulate the receptors on the immune cells that can detect cellular stress and danger signals.

The initial development of adjuvants was not able to produce clinical results due to their poor tolerance and reactogenic nature. Due to the characterizations of malaria and HIV vaccines, the rationale behind combining different adjuvants has emerged [173]. This could result in a more effective and diverse immune response. The combination of these adjuvants utilized a delivery system that included alum, emulsions or liposomes, as well as a variety of immune stimulating agents, such as dsRNA, bacterial MPLA or *Quillaja saponins* [173]. The new generation of cancer treatment systems were developed based on validated delivery models and pharmaceutical principles [134]. The delivery system for vaccines concentrates on the activation of the immune signal, which can be focused on the antigen formulation [174,175]. The AS01 vaccine, which utilized a liposome-based adjuvant system, provided better protection than the similar formulation used for the AS02 vaccine [66,176]. A recombinant protein-based malaria vaccine candidate (RTS,S) was developed using the AS01 adjuvant system, which demonstrated that combining two immunopotentiators is needed to achieve its goal. The AS01 adjuvant contains both saponin QS-21 and MPLA. Studies have shown that, when QS-21 and MPLA are combined, they activate an innate immune response, which further boosts the adaptive response [174,175,176]. The AS01 adjuvant system can stimulate the T-cell response in humans in response to various vaccine antigens. This is due to the synergistic effect of various immunopotentiators that incorporates with MPLA and QS-21. The results of these studies provide new directions in developing novel adjuvant systems [174].

The rationale behind most of the adjuvants used in vaccines has been confirmed by the data collected in clinical studies [66,174,175,176]. Adjuvants are usually used to stimulate a response in the immune system by draining the stimulated cells into the lymph nodes. This process helps boost the body’s adaptive immunity against pathogens. The activation of danger signal and PAMP pathways can promote the recruitment of various effector cells, such as B and T cells, and the activation of APCs. Studies have shown that the depletion of activated APCs can be used to stimulate the production of T cells that are specific for vaccine antigens. The antigen-specific T-cells’ phenotypes will be determined by the nature of the immune signal’s activation. The growth of CD4^+^ T cells can be stimulated by the secretion of interleukin-18 by subcapsular macrophages [177,178]. In addition, the secretion of certain cytokines by stimulated APCs can promote the growth of T cells that are capable of producing a type of immune response known as a follicular-helper phenotype [179]. The use of an adjuvant system that contains TLR ligands can enhance the quality and arrangement of antigen-specific T cells’ clonal repertoire. It can also stimulate the growth of these cells with enhanced TCR affinity [180].

Matrix-M is an important adjuvant developed by Novavax, Inc. This substance is derived from the bark of the Soapbark tree. Matrix-M adjuvant is utilized in formulating an anti-SARS-CoV-2 drug, which contains a recombinant protein. The COVID-19 vaccine is currently undergoing a clinical trial in the first phase. Moreover, the matrix-M adjuvant can stimulate the production of antibodies with high neutralizing capabilities and induce robust T-cell responses, rendering different strains of virus incapable of damaging them [181,182]. It has been known that certain saponins, such as QS-21, induce a high-antibody titre and an OVA-specific cytotoxic T-cell response by activating the caspase 1 in the subcapsular sinus. They also activate the tyrosine–protein kinase SYK by destabilizing the lysosome [183]. Saponins, such as matrix-M, play a significant role in the development of the innate immune system, which trigger both cell-mediated and humoral immune responses. The exact mechanism of action of this adjuvant is still unclear. This adjuvant mechanism of action can be defined by utilizing a system biological approach for vaccines [88]. The rationale and mechanistic approach are related to the acceptability and effectiveness of adjuvanted vaccines in human. This information can be useful in helping to develop a protocol that will allow clinicians and non-clinical models to evaluate and compare the various types of adjuvant systems. A study revealed that the effects of five different vaccines on CD4^+^ T cells were not able to change their phenotypes [184]. It is not always accurate to assume that the quality of an individual’s immune response is affected by adjuvants.

## 8. Nanoparticles as a Tool for Vaccine Delivery

Due to the limitations of vaccines, they are unable to precisely target the tissue they are designed to release, which leads to the indiscriminate distribution of vaccines in the host cells. Cells also receive large concentrations of drugs due to their position within the host cells [185]. This issue can be solved by using nanoparticle-based drug delivery systems. Vaccine delivery is one of the main applications of NPs, and they play a vital role in the development of new vaccines [186]. In the delivery of vaccines, it is important that the drugs are controlled and delivered to the target sites. Active and passive targeting are the primary two main approaches of targeting by NPs. The process of targeting involves the delivery of a vaccine precisely to a specific cell population to avoid damaging other organs. Non-targeting NPs undergo passive targeting as they do not have the targeting molecules. In contrast, active targeting involves the addition of a few targeting agents, which can be utilized to transfer to the cell’s surfaces. For instance, targeted delivery can be achieved by coating therapeutic particles with antibodies, which can recognize a specific antigen receptor in dendritic cells [187,188]. Vaccines can only be administered using super-paramagnetic oxide nanoparticles, which have a diameter ranging from 1 to 30 nm. Due to their unique magnetic domain, these particles can be utilized in the field of magnetic targeting. The ability to carry different biomolecules with varying surface functional groups and low cost of production are some of the main properties of iron oxide nanoparticles [189,190]. Traceability of the vaccines’ NPs can be a powerful advantage when it comes to delivering them in different ways. For instance, in combination with other antigens, inhalation or optical delivery can provide protection against multiple diseases [191].

Chemotherapeutics and targeted drugs can cause remission and lessen the overall burden of the tumour. Various cancer-related sequelae and resistance mechanisms hinder the development of a widely applicable cancer treatment. Numerous free peptides and surface antigens have been identified as being produced by cancer cells. These distinctive antigens, or peptides, serve as a basis for the development of innovative cancer vaccines that can protect patients against certain tumours. The development of cancer vaccines potentiates the necessity for robust delivery systems to treat various diseases. Various medical conditions, including cancer, can be treated through the use of nanoparticles [192]. Nanoparticles can be used to deliver a vaccine or an adjuvant that can stimulate the body’s immune response against different deadly diseases. A wide range of vaccines and agents are utilized for treating various types of diseases. These can be modified to specifically target tumours through the modification of their composition or surface properties. Table 4 summarizes the various types of cancer vaccines that are currently available [193]. One of the most challenging factors in developing a new vaccine is the ability to deliver the antigens to certain cell populations, such as the NK cells and APCs. This is because it allows the development of an immunological response against the disease. The fragile properties of antigens make them vulnerable to degradation in the blood microenvironment. The rapid degradation of peptides and antigens can result in a loss of delivery to the host cells that causes an inadequate immune response [61]. There is an urgent need to develop novel delivery methods that can deliver antigens without attenuating bioactivity.

One of the ways to improve the effectiveness of antigen delivery is by utilizing nanoparticles. Due to their advantages, nanoparticles are being considered as potential delivery vectors for cancer vaccines. These include their ability to maintain their biological activity, improve their bioavailability and prevent antigen degradation. A wide variety of nanoparticle types can be utilized as delivery systems, such as polymeric materials, micelles, carbon nanostructures, mesoporous silica nanoparticles (MSNs), gold nanoparticles (AuNPs) and viruses [212]. The incorporation of specialized surface-targeting agents can enhance nanoparticle throughput and optimize their penetration into specific cellular organelles that mediate the innate immune response [213,214,215]. Active vaccines can be developed by combining various factors such as cell-penetrating peptides, immune stimulation and APC-specific epitopes [216].

## 9. Current Challenges and Future Developments

The challenges in presenting naive antigens and toxicity are some of the factors that can limit the development of vaccines. In addition, there are some challenging approaches used in the production of NPs. One of the challenges in creating nano-vaccines in sterile environments is the scaling-up process. New techniques for scaling up nano-vaccines have made it possible to overcome some of the obstacles. However, one of the most challenging aspects of this process is handling the scale-up in a sterile environment [217,218]. NPs can easily have access to a wide variety of organs and tissues in the body due to their small size. Through intradermal injections, the use of a nano-vaccine can cause various skin conditions. In addition, the administration of drugs through oral or nasal routes can cause respiratory issues. Nanostructured materials can penetrate the protective barrier around the brain, which could cause damage to the brain cells [219,220]. Studies conducted on rats revealed that the aggregation of NPs could lead to vascular thrombosis. The incorporation of NPs with adjuvants may allow these compounds to accumulate inside the host cells, raising concerns about long-term exposure. For instance, another study conducted on rats revealed that the quantum dots could stay in the animal’s body for up to two years [221]. Although ISCOMs can be used in various animal vaccines, the use of saponin-based adjuvants has been prohibited in humans due to their toxicity. Therefore, there are still concerns regarding the use of NPs in vaccines [221,222].

Currently, the use of nanoparticles for the delivery of vaccines is still in its early stages. Studies must demonstrate the rigor and validity of the reported findings and prove their applicability to the clinical setting. Although the physical properties of nanoparticles are known to influence biological interactions, their precise impact on these processes is still poorly understood. The rapid clearance of nanoparticles can result in their degradation and make therapeutic interventions ineffective. New methods are needed to improve the reproducibility of materials. This can be carried out through the development of new methods that are designed to address the various problems that affect the quality of materials used in medical and public healthcare applications. The design of nanoparticles should be able to resist protein binding, exhibit no toxicity and escape fast clearance. There is a need to balance nanoparticle modifications to ensure optimal self-surface clearance and vaccine delivery. Vaccines should be directed toward specific areas of targeted cells or sites of activity in order to deliver them effectively. Vaccines that are designed to be delivered through nanoparticles are expected to be either biodegradable or non-toxic and can be cleared from the body completely. Various hybrid delivery systems, such as those involving the use of polymer–lipid hybrid and organic nanoparticles, can be utilized to enhance the cellular immunity.

Clinically based and virus-like particles have made significant progress, and others are being licensed to use globally [223]. Some nanoparticle-based delivery methods are still in their early stages of development and have poor outcomes. A different hypothesis states that cancer biology has already triggered a process that can suppress the immune system. This is typically observed in drug-resistant cancer types. Although a vaccine based on nanoparticles can trigger an immunological response, its intensity is unsatisfactory. For patients with certain diseases, the activation or stimulation of their immune response may require a customized pattern that can adapt to their specific condition. The delivery of nanoparticles into a tumour site could have a compromised immune response in the patients. Studies show that increasing the production of Treg activity could decrease the level of antigen expression. This means that the potential therapeutic effects of nanoparticle-based vaccines would require a prolonged and personalized immune response. A simple research strategy is needed to take immune cells or a tumour microenvironment and test the effects of nanoparticle therapy in different cancer types. The prognosis provided by humoral or ex vivo cellular responses can serve as a valuable reference point in the development of new nanoparticle products.

## 10. Conclusions

The field of adjuvant research has been continuously improving over the years. Currently, there are only a few vaccine adjuvants approved. Through a combination of iterative design and high-throughput screening, we will be able to identify and develop effective adjuvants that can enhance the immune response in the future. Fundamental knowledge of innate immunology continues to rise, allowing us to identify the optimal approach to developing vaccines. This knowledge can help us identify novel pathways and modify the existing signaling events. Through these methods, researchers could gain ultimate control over the activation of the innate immune response, allowing them to create new adjuvants that could result in better outcomes, such as unique T-cell polarization or decreased vaccine reactions.

However, the use of nanoparticles can help in enhancing the presentation of antigens and stimulating robust immune responses for effective immunization. One of the main factors that has prompted the development of vaccines is the use of virus-like or virus-modified nanoparticles. For instance, the success of HPV vaccines has encouraged the development of other types of vaccines by incorporating multiple or structural antigens, which can stimulate an immunological response and fight against cancer cells. Natural nanoparticle mimics have potential applications as a vaccine delivery mechanism. They can take advantage of their native functions, such as circulation and cellular recognition, and avoid the clearance mechanisms that are typical of nanoparticle systems. One of the biggest challenges in adopting nanoparticle systems is fast clearance. The mechanistic pathways that lead to the excretion of nanoparticles from tissues and cells after cellular uptake are still under investigation. Through the modulation of their surface properties, such as shape, composition and size, these nanoparticles can enhance the immune response against various diseases. In the future, the use of nanoparticles in clinical settings should be based on robust, reproducible and inexpensive methods for large-scale production.

## Figures and Tables

**Table 1:** Benefits and limitations of using different nanoparticle types for vaccine development.

Nanoparticles	Benefits	Limitations	Ref.
Gold	Low cytotoxicity Control the size and diameter Increased uptake because of ionic interaction with blood–brain barrier (BBB)	Need coating Non-biodegradable	[42]
Poly (lactic-co-glycolic acid) (PLGA)	Prolonged release of antigen Biodegradable Adjustable surface modifications FDA approved and non-toxic	Scale-up Antigen burst releases Degradation of antigen Self-aggregation may impact brain delivery	[43,44]
Lipid based	Biodegradable Wide size range Hydrophobic or hydrophilic cargo Antigen encapsulated or on surface FDA approved and non-toxic	Reproducibility issues Degradation by oxidative stress Costly to produce	[45,46]
Polystyrene	No cytotoxicity Biocompatible Wide size range Readily available	Non-biodegradable	[47]
Calcium based	Easy surface modification Low cytotoxicity	Limited data available Limited degradability	[48]
Quantum dot	High stability Produce fluorescence	High cytotoxicity Non-biodegradable	[49]
Self-assembling protein nanoparticles (SAPNs)	Biodegradable Repetitive presentation	Limited study available Complex synthesis and design	[50]
Superparamagnetic iron oxide nanoparticles (SPIONs)	Good magnetic property Biodegradable Control the size Approved by FDA	Stability issues Need coating	[51]
Immunostimulating complex or ISCOM	Readily available Natural adjuvant Biodegradable Scalable Well-tolerated	Single size Limited encapsulation	[52]
Polyethylenimine/poly-γ-glutamic acid (PEI/ɣ-PGA)	Small size Use for DNA vaccine	Limited data available	[53]
Lipid nanoparticles (LNPs)	No bio-toxicity High physical stability Potential vaccine adjuvant activity in mRNA vaccines High targeting qualities through ligand functionalization	Low drug load efficiency In vivo instability Short blood circulation time Toxicity concerns	[54]

**Table 2:** Adjuvant classification based on their primary mechanisms of action.

Groups of Adjuvants	Adjuvants Types	Proposed Mechanisms	Ref.
*Mucosal adjuvants*	Heat-labile enterotoxin (LTK3 and LTR72) Chitosan (CS) Cholera toxin (CT) Alginate	Increase the expression of MHC class II and costimulatory molecules to improve the antigen-presenting ability of APC. Improving the targeting of antigen-presenting cells, encouraging macrophages to release associated inflammatory factors and controlling the Th1/Th2 tendency to control the immune response. Immunomodulatory activity of CTB may have an underlying mechanism involving induction of MPK1 expression. Prolong a release of antigen and increase the immunogenicity more than traditional vaccines as hydrophilic carriers.	[111,112,113,114]
*Combined adjuvants*	AS01, AS02, AS03 and AS04	MPL, a strong agonist of the toll-like receptor (TLR) 4, is present in AS01, AS02 and AS04. The purpose of AS01, which includes MPL, QS-21 and liposomes, is to further enhance the CD8C T cells response. AS02 contains MPL and QS-21 in an emulsion oil-in-water solution, which triggers strong T-cell responses and humoral responses. AS03, an oil-in-water emulsion containing alpha-tocopherol, or vitamin E, boosts immunity by the activation of human monocytes and macrophages. AS04 stimulates a Th1 biased immune response, which is evaluated in vaccines against viral infections.	[66,115,116,117,118]
*Immune potentiators* TLR1/2 agonists TLR3 agonists TLR4 agonists TLR5 agonists TLR7/8 agonists TLR9 agonists	L-pampo, MALP-2, Pam2CSK4 and Pam3CSK4 Poly(I:C) (polyinosinic: polycytidylic acid) Poly-ICLC Monophosphoryl lipid A (MPL) Flagellin Imiquimod (R837; 1-(2-methylpropyl)-1H-imidazo [4,5-c]quinolin-4-amine) and resiquimod (R848, 4-amino-2-(etoximetil)-a, a-dimethyl-1H-imidazo [4,5-c]quinoline-1-ethanol) CpG ODNs	L-pampo stimulates the recruitment of dendritic cells into lymph nodes, where they activate T lymphocytes that are specific for the antigen. MALP-2 stimulates the NF-kB pathway to cause the release of IL-6, IL-8 and granulocyte-macrophage colony stimulating factor (GM-CSF) by amniotic mesenchymal cells. Toll-like receptor 2 ligand Pam2CSK4 activates platelet NF-kB and Bruton’s tyrosine kinase signaling to promote platelet–endothelial cell interactions. Induces secretion of pro-inflammatory cytokines and chemokines, which are capable of eliciting in vitro immune cell activation. Evidence suggests that MPL increases costimulatory molecules (B7-1 and B7-2) on monocytes, macrophages and dendritic cells. TLR5-expressing cells are activated by flagellin through either MyD88-dependent or -independent mechanisms. These activate immune cells via the TLR7/TLR8 MyD88-dependent signaling pathway, which triggers NF-kB activation in cells expressing murine TLR8. Stimulate the innate immune system by binding to the cell’s TLR-9 receptors. The resulting innate immune response promotes the activation of an adaptive immune response in the presence of foreign antigens by producing Th1 and proinflammatory cytokines and chemokines.	[119,120,121,122,123,124]
*Delivery systems* Mineral salts Emulsions Microparticles	Aluminium salts Freund’s adjuvants (MF59) Virus-like particles Virosomes PLA/PLGA	In vivo activation of NLRP3 response, which is independent of TLR signaling. It increases the production of local chemokines and cytokines and encourages the recruitment of immune cells by increasing the expression of antigen-presenting cells. These are independent of NLRP3 response, but dependent on ASC and MyD88 response. They increase the production of local chemokines and cytokines and encourage the recruitment of immune cells by increasing the expression of antigen-presenting cells. Due to their inherent immunogenicity, which can stimulate both cellular and humoral immune responses, they are safe and efficient immune stimulators and play significant roles in the development of vaccines. They serve as antigen delivery vehicles, which bind with the APCs and induce receptor-mediated endocytosis. They also escape endosomal degradation and increase the expression of antigen-presenting cells. PLGA particles can be taken up by DCs and macrophages through macropinocytosis, clathrin-dependent receptor-mediated endocytosis and phagocytosis, demonstrating their strong phagocytic abilities.	[64,125,126]

MHC class II, major histocompatibility complex class II; APC, antigen-presenting cell; CTB, cholera toxin subunit B; MPK1, mitogen-activated protein kinase 1; MPL, monophosphoryl lipid A; TLR, toll-like receptor; QS-21, *Quillaja saponaria* 21; MALP-2, macrophage-activating lipopeptide-2; NF-kB, nuclear factor kappa B; GM-CSF, granulocyte-macrophage colony stimulating factor; TLR5, toll-like receptor 5; PLGA, poly(lactic-co-glycolic acid).

**Table 3:** Vaccine adjuvants approved for human use. (Reprinted with permission from [170]).

Vaccine Adjuvants	Formulations	Trade Name	Disease(s)	Description	Licensed (Year)
Alum	Aluminum as mineral salt	Daptacel, Twinrix, Gardasil, Bexsero and Prevnar 20	Diphtheria, pertussis, tetanus, hepatitis A and B, inactivated poliomyelitis vaccine, human papilloma virus, meningococcal and pneumococcal	Hydroxide, phosphate or hydroxyphosphate sulfate salt particles that are insoluble. Salt-induced antigen adsorption; humoral immunity modulation; Th2 type of immunological response; increases in inflammation.	1962
Virosome	Liposome	Epaxal	Influenza and hepatitis A	This activates the T-cell response by promoting the uptake of antigen by both APCs and B cells. Modulates cellular and humoral immune responses.	2000
AS03	Oil-in-water emulsion	Pandemrix, Arepanrix and aTIV	Influenza (pandemic)	This stimulates the production of certain cytokines and the recruitment of immune cells. Modulates cellular and humoral immune responses.	2009
MF59	Oil-in-water emulsion	Fluad	Influenza (both seasonal and pandemic)	Promotes the recruitment of APCs and their activation, stimulates immune cells to take up antigens and migrate to lymph nodes, and modulates humoral and cellular immune responses.	1997
AS04	Alum-adsorbed TLR4 agonist	Cervarix and Fendrix	Hepatitis B virus and human papilloma virus	Enhances APC maturation and induces Th1-type immunological responses by stimulating TLR-4. Modulates humoral and cellular immune responses.	2005
RC-529	Synthetic TLR4 ligand adsorbed to aluminum hydroxide	Supervax	Hepatitis B virus vaccine approved in Argentina	Increases the expression of cell-surface costimulatory molecules and receptors, cytokines and chemokines by activating TLR4.	2004
Imiquimod	Synthetic TLR7 agonist	Aldara	Genital and perianal warts, actinic keratosis	This induces T-cell response by activating Langerhans cells, which then migrate to lymph nodes.	1997
Alhydroxiquim-II	Alum adsorbed to TLR7/8 agonist	COVAXIN	COVID-19	Two cellular receptors (TLR7/8) are activated by small molecules of Alhydroxiquim-II which migrate to lymph nodes and detach from alum.	2022
CpG ODN (1018 ISS)	Soluble TLR9 ligand (oligonucleotide) co-administered with HBV vaccine	Heplisav-B	Hepatitis B	Boosts humoral immunity, Th1 type immunity and CD8^+^ T-cell-mediated immunity.	2012
CpG ODN (1018 ISS)	Soluble TLR9 ligand (oligonucleotide) adsorbed to alum	CorbeVax	COVID-19	Increased humoral and cellular immunity with significant production of Th1-specific cytokines.	2022

TLRs, toll-like receptors; HBV, hepatitis B vaccine; APC, antigen-presenting cell.

**Table 4:** Various types of cancer vaccines and their clinical implementation.

Vaccine	Cancer Type	Description	Stage of Development	Mechanism	Ref.
Gardasil4/9 Cervarix—contain L1 proteins from different strains	Cervical	Allogenic	In clinical use	Mostly generates antibodies that are neutralizing against several human papillomavirus (HPV) strains	[194,195]
HEPLISAV-B hepatitis B surface antigen	Liver	Allogenic	In clinical use	Antibody response cytotoxic T lymphocyte (CTL)	[196]
CIMAvax-EGF	Lung	Allogenic	Clinical trial	Antibody	[197]
Neovax (personalized neoantigens)	Melanoma	Autologous	Clinical trial	CD4 and CD8	[198]
Sipuleucel-T (prostate acid phosphatase antigen (PAP))	Prostate	Autologous (antigen-presenting cells of patients incubated with PAP and GM-CSF)	In clinical use	T cell	[199,200]
CEA (carcinoembryonic antigen) Muc1 Peptide/DNA	Colorectal	Autologous or allogenic	Preclinical and in clinical trial	CTL response	[201,202,203]
Carbonic-anhydrase IX and HLA-A 0201/0206-restricted epitope peptide (HIG2-9-4) vaccine	Kidney	Allogenic/autologous	In clinical trials	Increase in interferon (IFN) responses to CTL	[204,205]
BCG (Bacillus Calmette–Guérin)	Bladder	Autologous (mainly used against tuberculosis)	In clinical use	BCG antigen internalization and antigen-specific CD8 and CD4 T cell activation	[206,207]
IDH1(R132H)-specific peptide vaccine DCVax^®^-L (dendritic-cell-based personalized vaccine)	Brain	Allogenic (isocitrate dehydrogenase1, becomes mutated in gliomas) Autologous	Phase 1 Phase 3	Specific immune response against the mutated protein Specific immunological response to the mutated protein	[208,209]
Her 2 directed cellular/DNA/viral and telomerase reverse transcriptase	Breast	Autologous or allogenic	CTL activation towards mutations in overexpressing breast cancer cells and activation of immune response	Phase 1/2	[210,211]

CTL, cytotoxic T lymphocyte; CEA, carcinoembryonic antigen; IFN, interferon; BCG, Bacillus Calmette–Guérin.
